# Fabrication of Two-Layer Microfluidic Devices with Porous Electrodes Using Printed Sacrificial Layers

**DOI:** 10.3390/mi15081054

**Published:** 2024-08-22

**Authors:** Kosuke Ino, An Konno, Yoshinobu Utagawa, Taiyo Kanno, Kazuyuki Iwase, Hiroya Abe, Hitoshi Shiku

**Affiliations:** 1 Graduate School of Engineering, Tohoku University, 6-6-11-604 Aramaki-aza Aoba, Aoba-ku, Sendai 980-8579, Japan; 2 Graduate School of Environmental Studies, Tohoku University, 6-6-11-604 Aramaki-aza Aoba, Aoba-ku, Sendai 980-8579, Japan; 3 Institute of Multidisciplinary Research for Advanced Materials, Tohoku University, Sendai 980-8577, Japan; 4 Frontier Research Institute for Interdisciplinary Sciences, Tohoku University, Aramaki-aza Aoba 6-3, Aoba-ku, Sendai 980-8578, Japan

**Keywords:** microfluidic device fabrication, sacrificial layer-based 3D printing, Pluronic F-127, electrochemical measurement

## Abstract

Two-layer microfluidic devices with porous membranes have been widely used in bioapplications such as microphysiological systems (MPS). Porous electrodes, instead of membranes, have recently been incorporated into devices for electrochemical cell analysis. Generally, microfluidic channels are prepared using soft lithography and assembled into two-layer microfluidic devices. In addition to soft lithography, three-dimensional (3D) printing has been widely used for the direct fabrication of microfluidic devices because of its high flexibility. However, this technique has not yet been applied to the fabrication of two-layer microfluidic devices with porous electrodes. This paper proposes a novel fabrication process for this type of device. In brief, Pluronic F-127 ink was three-dimensionally printed in the form of sacrificial layers. A porous Au electrode, fabricated by sputtering Au on track-etched polyethylene terephthalate membranes, was placed between the top and bottom sacrificial layers. After covering with polydimethylsiloxane, the sacrificial layers were removed by flushing with a cold solution. To the best of our knowledge, this is the first report on the sacrificial approach-based fabrication of two-layer microfluidic devices with a porous electrode. Furthermore, the device was used for electrochemical assays of serotonin and could successfully measure concentrations up to 5 µM. In the future, this device can be used for MPS applications.

## 1. Introduction

Porous and thin membranes are widely used in microphysiological systems (MPS) [1]. In MPS using membranes, cells are cultured on both sides of the membrane to mimic the interfaces of tissues and organs, such as the gut, performing in vivo functions. Porous membranes have also been incorporated into microfluidic devices. Because two-layer microfluidic devices have interconnected top and bottom channels, functional organs can be fabricated by applying shear stress to the cells. Recently, electrodes have been placed on membranes to monitor the cellular activity in situ [2,3,4]. In one of these studies, a Au electrode was prepared on a polyethylene terephthalate (PET) membrane by sputtering, and vascular endothelial cells were cultured on the porous electrode to prepare vascular models. Nitric oxide released from the vascular models was monitored in situ using the electrode [2]. Generally, such microfluidic devices are prepared with soft lithography using polydimethylsiloxane (PDMS) [5].

Microfluidic devices are useful tools for bioapplications such as cell culture [6] and cell sorting [7]. Three-dimensional (3D) extrusion printers have recently been used to fabricate microfluidic devices directly because of their flexibility. 3D extrusion printers are widely used to construct 3D organs and tissues [8]. Generally, cell-laden hydrogels are directly bioprinted. In contrast, materials such as sugar [9,10], triblock copolymers of polyethylene–polypropylene–polyethylene (Pluronic F-127) [11,12,13], and water-soluble polyvinyl alcohol [14] have been used as sacrificial templates for biofabrication. In this approach, the sacrificial layers were three-dimensionally printed using a 3D extrusion printer [11] and viscous fingering [12]. In addition to the 3D extrusion printer, an inkjet printing technique was reported to prepare a sacrificial layer [15]. After covering with hydrogels or PDMS, the layers were removed to prepare the microfluidic channels. As this fabrication process is flexible, sacrificial template-based 3D bioprinting is a promising approach for fabricating organs such as vasculature [16]. Among the available materials, Pluronic F-127 is a promising material that can be used for cell culture applications [17]. Because the solution of Pluronic F-127 exhibits temperature-responsive behavior and is highly viscous at room temperature, Pluronic F-127 is a printable material. The 3D printing of sacrificial Pluronic F-127 layers has been employed to fabricate vascular models [11,18]. Although this sacrificial layer-based approach has been used to fabricate monolayer microfluidic channels, there have been no reports on the fabrication of two-layer microfluidic devices with porous electrodes. Therefore, in this paper, we present a novel fabrication process for a two-layer device using 3D printing with Pluronic F-127 as the sacrificial layers. After the fabrication was completed, the device was used to electrochemically measure serotonin, which plays a crucial role in the gut [19].

## 2. Materials and Methods

### 2.1. Device Fabrication

The fabrication of the porous Au electrodes is described in our previous study [2]. Briefly, Ti, Pt, and Au were sequentially sputtered on track-etched PET membranes (pore size: 8 μm, pore density: 6 × 10^4^/cm^2^, thickness: 16 μm, it4ip, Belgium).

The sacrificial layers as microfluidic channels were designed using CAD software (Figure S1). Although smaller constructs can be printed, the repeatability is not good. Therefore, the channel size of 1 mm height and 2 mm width was selected. Pluronic F-127 (Sigma Aldrich, USA) was dissolved in deionized water to prepare a 40% (*w*/*v*) solution as an ink and stored at 4 °C. The ink was incubated at 25 °C in a thermostatic bath for 30 min and then introduced into a 22G syringe. The ink was printed at 60 mm/s and 85 kPa using a 3D extrusion bioprinter (INKREDIBLE, Cellink, Sweden). According to the user manual of this printer, the positioning precisions of XY and Z are 10 and 2.5 µm, respectively, and the layer resolution is 100 µm. As the syringe (inner diameter: 410 µm) was used, the minimum resolution is expected to be approximately 400 µm.

**Figure 1:**
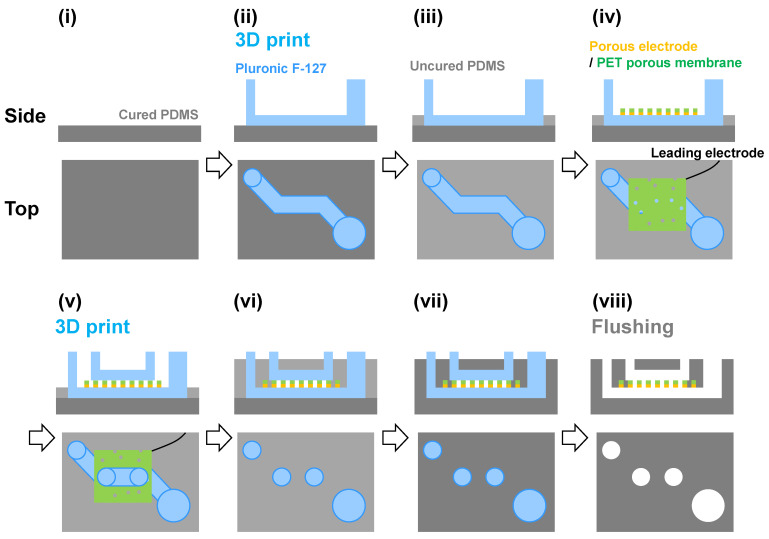
Schematic of the fabrication process of the two-layer microfluidics device with the porous electrode. Pluronic F-127 was used as the sacrificial ink. (**i**) Preparation of a cured PDMS film as the 1st layer. (**ii**) Printing of the sacrificial layer of the Pluronic F-127 ink for the bottom channel. (**iii**) Pouring the uncured PDMS up to the height of the bottom channel. (**iv**) Setting the porous Au electrode on the device. The PET porous membrane side is at the top. (**v**) Printing of the sacrificial layer of the Pluronic F-127 ink for the top channel. (**vi**) Pouring the uncured PDMS to fill the entire area. (**vii**) Curing the PDMS layers. (**viii**) Flushing out the sacrificial layers.

Figure 1 illustrates the fabrication process. First, 2 g of PDMS before curing was introduced into a 60 mm dish and cured to prepare the 1st PDMS layer. The Pluronic F-127 ink was then three-dimensionally printed to form the bottom channel (Figure S1). The uncured PDMS was introduced to prepare the 2nd PDMS layer. Next, a porous Au electrode was placed on the device with the electrode side facing the bottom. The Pluronic F-127 ink was then three-dimensionally printed for the top channel (Figure S1). These channels were covered with PDMS before curing. The device was vacuum-deaerated for 1 h, then incubated at 70 °C for 1 h. Finally, cold water or ethanol was introduced into the channels to remove these sacrificial layers. The resulting area at the bottom channel for electrochemical reactions was approximately 35 mm^2^. The device was then removed from the dish to complete the fabrication.

For investigating the effects of printing on fouling electrodes, a porous Au electrode was electrochemically characterized before incorporating it into the device. The electrode was covered with insulating tape, resulting in a 50 mm^2^ area for electrochemical reactions.

### 2.2. Cyclic Voltammetry

Cyclic voltammetry (CV) of ferrocenemethanol (Tokyo Chemical Industry Co., Ltd., Japan) and serotonin (Wako, Japan) in phosphate-buffered saline (PBS) was performed using a potentiostat (HA1010mM4, Hokuto Denko, Japan). A porous electrode was used as the working electrode before and after incorporation of the microfluidic device. The working Ag/AgCl (sat. KCl) reference and Pt wire counter electrodes were placed in the sample solution (Figure S2).

## 3. Results and Discussion

**Figure 2:**
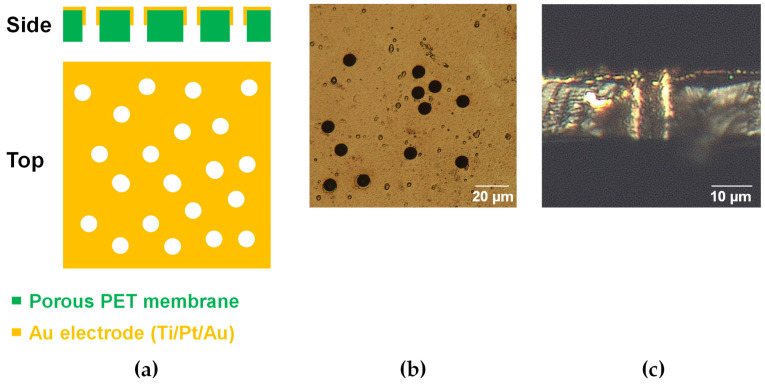
Porous Au electrode. (**a**) Schematic illustration and bright-field images of the (**b**) top and (**c**) cross-section of the porous electrodes.

Figure 2a shows an outline of the porous electrode. Figure 2b,c show bright-field images of the porous electrodes before being incorporated into the device. No cracks are observed on the porous electrodes (Figure 2b). The cross-sectional image shows that the Au layers have penetrated the straight pores (Figure 2a,c).

**Figure 3:**
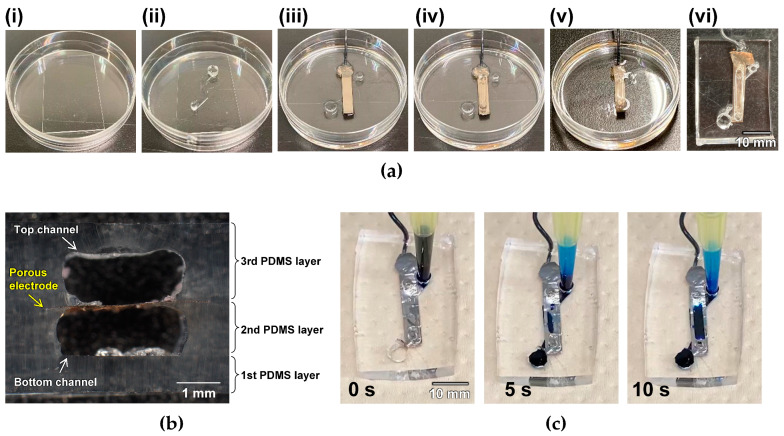
Two-layer microfluidic devices with porous electrodes using the proposed strategy. (**a**) Fabrication process. (i) Setting the 1st PDMS layer. (ii) Printing the bottom channel using the Pluronic F-127 ink. (iii) Preparing the 2nd PDMS layer and setting the porous electrode membrane. (iv) Printing the top channel using the Pluronic F-127 ink. (v) Preparing the 3rd PDMS layer. (vi) Flushing out the sacrificial layers and cutting the device to be removed from the dish. (**b**) Cross-sectional image of the two-layer microfluidic device with the porous electrode. (**c**) Time-course images of the device at 0, 5, and 10 s after introducing the blue ink.

Subsequently, a two-layer microfluidic device with a porous electrode was fabricated using the 3D extrusion printer. Figure 3a shows the fabrication process. Owing to the high viscosity of Pluronic F-127, the 3D constructs were successfully printed as sacrificial layers above and under a porous electrode. The results indicate that PDMS did not affect the Pluronic F-127 ink constructs. As the Pluronic F-127 pole construct with a 2 mm diameter and 3 mm height, as shown in Figure S1, was successfully printed, the limitation of the aspect ratio was over 1.5 when this size of constructs was fabricated. The sacrificial layers were successfully flushed with a cold solution. The cross-sectional images supported the fact that the sacrificial layers were removed completely (Figure 3b). The height and width of each channel were approximately 1 and 2 mm, respectively. The shapes of the microfluidic channels were approximately the same as those in the design (Figure S1), indicating that this strategy can be used for fabrication based on the sacrificial layers. Next, the repeatability of the printing was investigated. Briefly, the sacrificial layers of Pluronic F-127 were printed several times, and PDMS channels were fabricated using the proposed method. The cross-sections were then observed. As shown in Figure S3, the height was almost constant even though the printing was repeated, indicating that the repeatability of the height was good. However, the repeatability of the width and shape was a little low (Figure S4). The low repeatability may be caused by the manual operations for preparing second PDMS layers and setting porous electrodes on them.

Next, a blue ink solution was introduced through the bottom channel inlet to check for leakage. Figure 3c shows that the ink flows from the inlet to the outlet of the bottom channel without leakage. In addition, the ink flows from the bottom to the top channel through the porous electrode, indicating that the pores maintain clear penetration after the fabrication process. Thus, this printing approach can be applied to the fabrication of two-layer microfluidic devices with a porous electrode.

**Figure 4:**
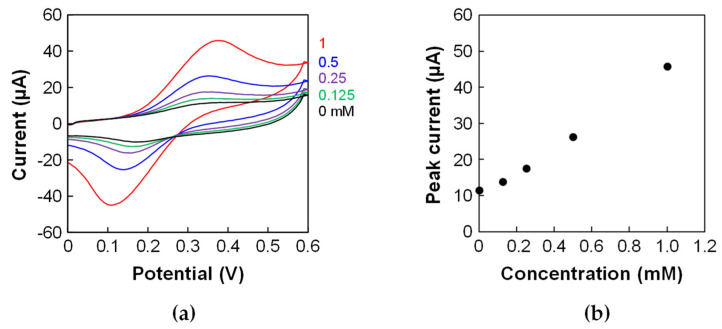
CV of ferrocenemethanol using the porous electrode in the device. (**a**) Cyclic voltammograms of 0, 0.125, 0.25, 0.5, and 1 mM ferrocenemethanol in PBS with 0.1 M KCl. Scan rate: 100 mV/s. (**b**) Calibration plots using the peak currents of the cyclic voltammograms.

The porous electrode in the device was electrochemically characterized using CV with ferrocenemethanol. The Δ*E*_p_ in the cyclic voltammograms was large (Figure 4a) compared to that of the porous electrode before incorporating it into the device in the previous study [2]. Furthermore, the Δ*E*_p_ was larger than that of the porous electrode in the microfluidic device fabricated without the sacrificial-layer approach in the previous study [2]. This result indicates that the porous electrode was slightly fouled during the fabrication process and that fouling affected the electron transfer during the electrochemical reaction. However, the calibration curve showed a linear relationship between the concentration and peak current (Figure 4b). These results indicate that the device can be used for quantitative assays.

**Figure 5:**
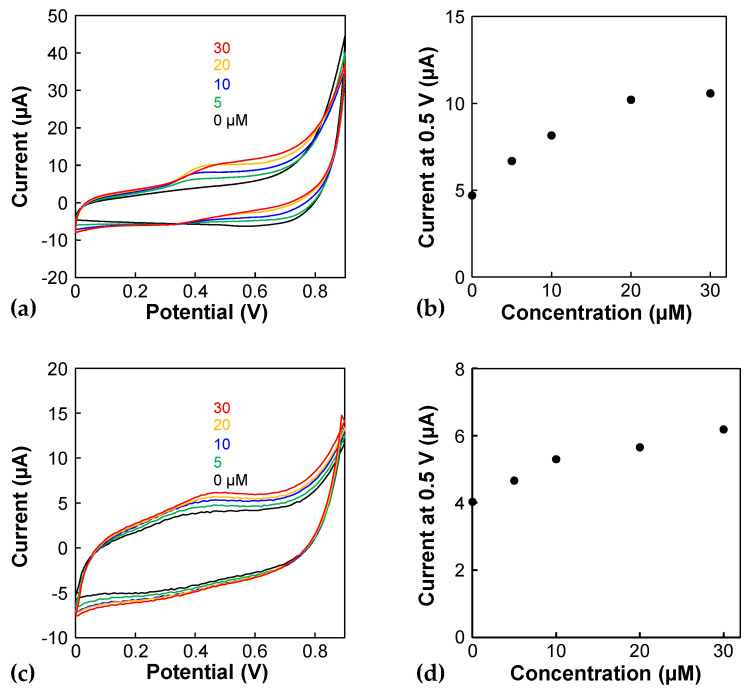
CV of serotonin using the porous electrode (**a**,**b**) before and (**c**,**d**) after incorporating it into the device. (**a**,**c**) Cyclic voltammograms of 0, 5, 10, 20, and 30 µM serotonin in PBS. Scan rate: 100 mV/s. The results were obtained from the 2nd CV scan. (**b**,**d**) Calibration plots of the cyclic voltammograms using the currents at 0.5 V.

Finally, the serotonin concentration was measured electrochemically. Figure 5a shows the cyclic voltammograms obtained using the porous electrode before its incorporation into the device. The oxidation peak currents of serotonin appear at approximately 0.45 V, and the oxidation currents depend on the serotonin concentration. The calibration curve shows linearity ranging from 0 to 20 µM. However, the oxidation currents become saturated at higher concentrations (Figure 5b). Serotonin could have been absorbed onto the porous Au electrode and reacted electrochemically, leading to saturation due to the limitation of the active area. Next, three independent experiments were conducted for 0 and 5 µM solutions to evaluate the detection of limit. The currents of the 0 and 5 µM solutions were 5.12 ± 1.28 and 7.92 ± 0.425 µA (*n* = 3), and a Student *t*-test shows a significant difference (*p* < 0.05), indicating that the detection of limit is less than 5 µM. Figure 5c shows cyclic voltammograms obtained using the porous electrode in the device. The oxidation currents per charging current decrease drastically compared to those obtained using a porous electrode before incorporation into the device, indicating that the electrode in the device might be fouled. However, the oxidation peak of serotonin was observed in the cyclic voltammograms (Figure 5c) and on the calibration curve, even at a concentration as low as 5 µM (Figure 5d). As the serotonin concentration of the supernatant of a culture medium, in which a pancreatic delta cell line was cultured, was approximately 5 µM [3], the present device can be applied to the serotonin analysis of cells. However, the serotonin concentration using a monolayer of enteroendocrine cells derived from human primary intestinal stem cells was much lower than this concentration [20], suggesting that the sensitivity of the present sensors should be improved. Electrode modifications and differential pulse voltammetry are promising approaches for this purpose. For example, a previous study using carbon nanotube-modified electrodes detected 0.1 µM serotonin [21]; this material is promising for improving the sensitivity.

Although a simple electrode was prepared on the porous membrane in the present study, if microelectrodes are prepared there, the device can be applied to further bioapplications. For example, previous electrode devices are used to manipulate bioparticles for bioanalysis, cell sorting and tissue engineering using electrophoresis [22], electrorotation [23], electrofusion [24], and dielectrophoresis [25,26,27,28,29,30,31] techniques. To pack many sensing and reaction areas into a small device, 3D-patterned microelectrode arrays were prepared [23,24,29,30,31,32]. Thin and porous membranes are useful for preparing such microelectrodes because both sides can be used for the preparation of these electrodes. In the future, 3D-patterned porous electrode array devices can be applied to cell analysis and tissue engineering applications.

## 4. Conclusions

This paper proposes a novel fabrication process for two-layer microfluidic devices with porous electrodes. Pluronic F-127 was used as a sacrificial layer to create microchannels on a Au electrode. To the best of our knowledge, this is the first report of the fabrication of this type of device. Serotonin concentrations were measured using a porous membrane in the device. Although the sensitivity should be improved for the analysis of MPS using intestinal stem cells, a proof-of-concept of the fabrication process was successfully achieved. In conventional devices fabricated with sacrificial layers, there is no porous membrane in a two-layer device. In contrast, the present device has a porous membrane, and therefore the device can be applied to the fabrication of functional organs by culturing cells at both sides of the membrane, which is advantageous to conventional devices. In the future, the electrode surface will be modified for highly sensitive assays, and the improved device will be used for the evaluation of MPS, including gut models.

## Data Availability

The raw data supporting the conclusions of this article will be made available by the authors on request.

## Supplementary Materials

The following supporting information can be downloaded at https://www.mdpi.com/article/10.3390/mi15081054/s1, Figure S1: Outline of the printed top and bottom channels in CAD; Figure S2: Setup for cyclic voltammetry. WE: working electrode. RE: reference electrode. CE: counter electrode. Figure S3: Repeatability of the printing. Figure S4: Cross-sectional images of successful and failed devices.
