# Supplementary material for: Fabrication of Two-Layer Microfluidic Devices with Porous Electrodes Using Printed Sacrificial Layers
Source: Micromachines (Basel). 2024 Aug 22;15(8):1054. doi: 10.3390/mi15081054 (PMC11356774; doi:10.3390/mi15081054)
Supplement: Supplementary file 1 [file micromachines-15-01054-s001.zip › micromachines-3164495-supplementary.pdf]

micromachines

Supporting Information

Fabrication of Two-Layer Microfluidic Devices with Porous Electrodes Using Printed Sacrificial Layers

Kosuke Ino [1,*], An Konno [2], Yoshinobu Utagawa [1], Taiyo Kanno [1], Kazuyuki Iwase [3], Hiroya Abe [1,4] and Hitoshi Shiku [1,2,*]

[1] Graduate School of Engineering, Tohoku University, 6-6-11-604 Aramaki-aza Aoba, Aoba-ku, Sendai 980-8579, Japan
[2] Graduate School of Environmental Studies, Tohoku University, 6-6-11-604 Aramaki-aza Aoba, Aoba-ku, Sendai 980-8579, Japan
[3] Institute of Multidisciplinary Research for Advanced Materials, Tohoku University, Sendai 980-8577, Japan
[4] Frontier Research Institute for Interdisciplinary Sciences, Tohoku University, Aramaki-aza Aoba 6-3, Aoba-ku, Sendai 980-8578, Japan
* Correspondence: kosuke.ino@tohoku.ac.jp (K.I.); hitoshi.shiku.c3@tohoku.ac.jp (H.S.)

Contents

-Figure S1
-Figure S2
-Figure S3
-Figure S4

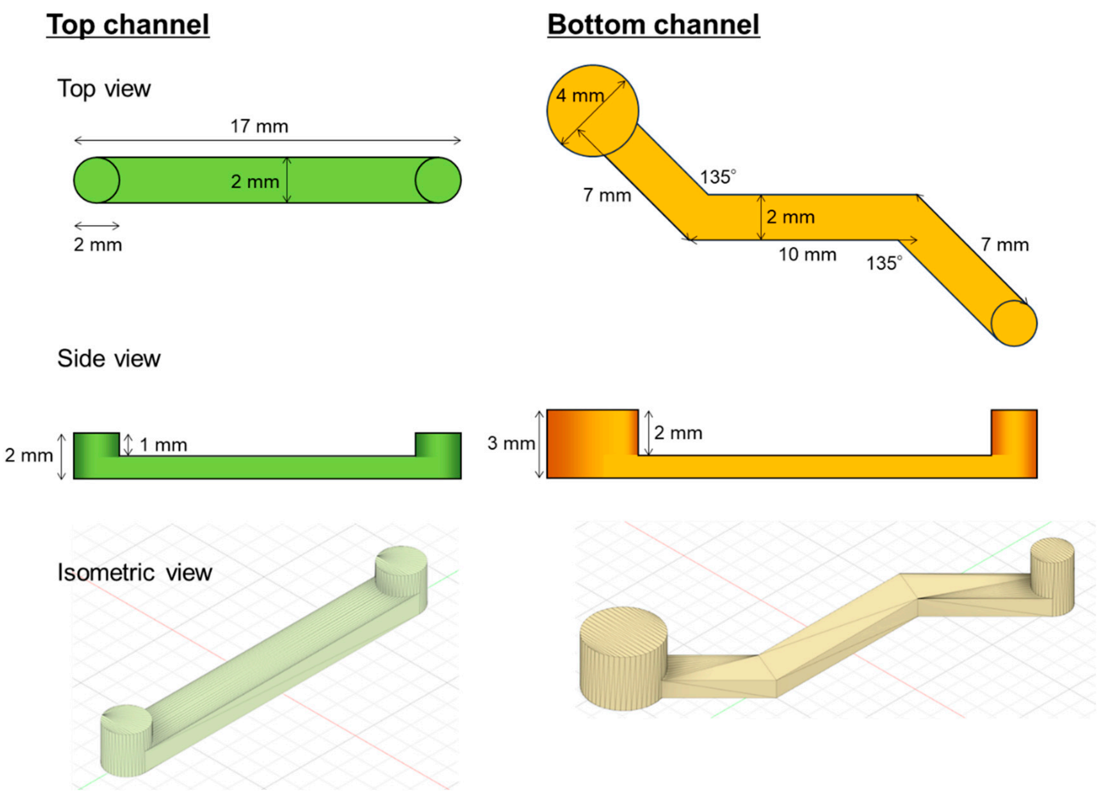

Figure S1. Outline of the printed top and bottom channels in CAD.

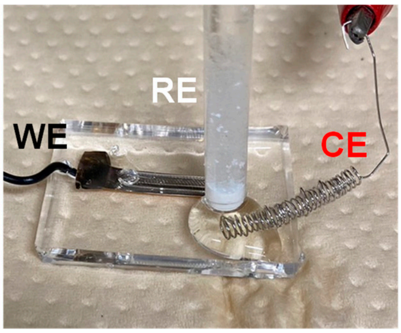

Figure S2. Setup for cyclic voltammetry. WE: working electrode. RE: reference electrode. CE: counter electrode.

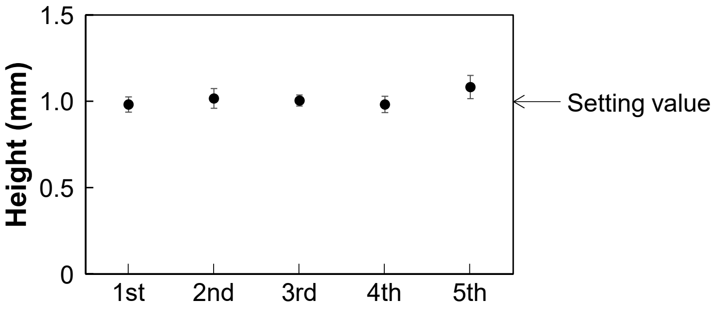

Figure S3. Repeatability of the printing. The sacrificial layers of Pluronic F-127 were printed on PDMS layers several times at 60 mm/s and 85 kP. After fabricating PDMS channels using the method described in the main text, the cross-sections were observed.

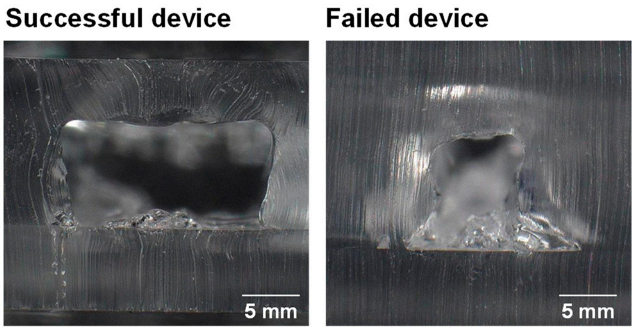

Figure S4. Cross-sectional images of successful and failed devices.
